# Evaluation of Primary Correction and Its Influencing Factors in Adolescent Idiopathic Scoliosis After Treatment with the Charleston Bending Brace as the Sole Intervention

**DOI:** 10.3390/life15030448

**Published:** 2025-03-12

**Authors:** Susanne Froehlich, Annett Klinder, Morris Stirn, Wolfram Mittelmeier, Katrin Osmanski-Zenk

**Affiliations:** Orthopedic Department, University Medicine Rostock, 18057 Rostock, Germany

**Keywords:** major curve, outcome, misalignment, conservative therapy, compliance

## Abstract

Background: All-day braces are predominantly used for the conservative treatment of adolescent idiopathic scoliosis (AIS). The Charleston Bending Brace is a pure nighttime brace. The aim of this study was to investigate the primary in-brace correction of the main curve of AIS when treated with the Charleston Bending Brace. Specifically, the factors influencing major curve correction were examined. Methods: The retrospective analysis included 97 patients with AIS who were treated between October 2010 and September 2020. Patients with secondary scoliosis or orthotic pretreatment were excluded. Standardized radiographs were used to determine the Cobb angle of the major and minor curves. Curve correction in relation to Lenke’s classification, the Risser stage, and rotation were assessed at four different time points (t0: before treatment, t1: 6–12 months, t2: 13–24 months, and t3: 25–36 months during treatment). Results: The average Cobb of the main curve at the beginning of the study was 25.7°. The night brace achieved excellent in-brace correction at t1, with nearly half of the patients (43%) showing a correction exceeding 80%. Curve localization, the Lenke type, and the Nash–Moe rotation significantly influenced initial in-brace curve correction at t1. At t2, there was also a significant in-brace correction of the initial Cobb by 93.0%. Similar improvements were observed at t3 for in-brace correction as well as without the brace (*p* < 0.031). Conclusions: The results of the study revealed good primary in-brace correction of the main curve of the AIS with the nighttime brace, which was at least equivalent when compared to values from the literature for the Chêneau brace. Also, while restricted to medium-term results due to our study limitations, the percentage of correction in out-of-brace data of our patients was similar to weaned 24 h brace patients.

## 1. Introduction

Adolescent idiopathic scoliosis (AIS) is a three-dimensional growth disorder that occurs in adolescence and results in permanent coronal curvature of the spine in the frontal plane with co-axial rotation and torsion of individual vertebral bodies [1]. The etiology remains largely unknown [2]. In terms of pathogenesis, there is a disproportion in the growth of the dorsal element and the vertebral body itself. Due to restricted vertebral height increase, the vertebral bodies begin to rotate to make more space for themselves [3]. The development of AIS is not only age- but also sex-dependent as it is more common in girls than in boys [4,5,6]. The prognosis of progression is determined by the Cobb angle as a measure of the major and minor curves of the scoliosis, the patient’s age, the Risser stage as a measure of the remaining growth reserve, and menarche [7,8,9].

An earlier onset of idiopathic scoliosis in adolescence is linked to a higher risk of progression of deformity [8]. The classification of AIS is based on age, curve localization, and the convexity of the main curve [10]. There are several classification systems for AIS [11]. The Lenke classification as the current standard and most commonly used system is an improvement of the King classification [12]. Apart from six basic classifications of curve location, it also includes the lumbar deviation from the frontal plane and the curvature in the sagittal profile. Treatment is primarily determined by the Cobb angle of the major curve [13]. Scolioses of less than 20° are monitored clinically at regular intervals, every 3–6 months, and radiographically if progression is suspected. Physiotherapy may also be initiated. Although there is no evidence of progression control by these measures, scoliosis-specific physiotherapy is part of the therapeutic concept [14]. If the main curve is 20–25° or higher, orthosis fitting is recommended before the end of the growth stage [5,15,16]. This therapeutic component of scoliosis treatment is the only non-surgical therapy for which there is scientific evidence (level II evidence: Cochrane Review) [17,18,19,20,21,22,23]. The orthoses are custom-made. The Chêneau or Boston brace is one of the " classical " day braces, or more precisely, a 24 h brace [23,24,25]. Part-time or night orthoses have been developed to reduce poor compliance and psychological stress [26]. Compared to other orthoses, the Charleston Bending Brace, which was introduced in 1989, showed fewer restrictions on the quality of life in terms of pain, sleep, and psychosocial factors. The aim is to increase the compliance by reducing the actual wearing time to night time.

**Figure 1:**
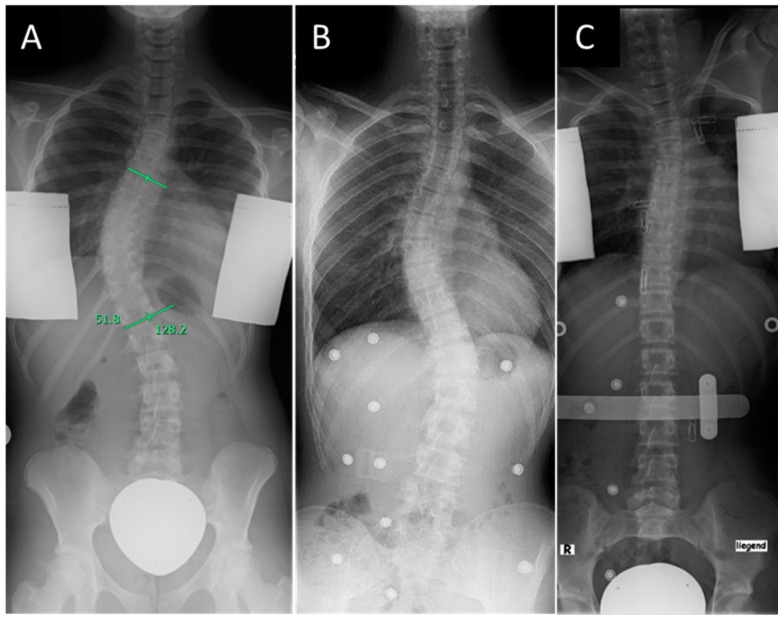
Right thoracic idiopathic scoliosis of Lenke type 1 identified in a standing radiograph (**A**), standing radiograph to assess the correction of the curve in the Chêneau corset (**B**), and supine radiograph to assess the correction of the curve in the Charleston Bending Brace (**C**).

It is the best alternative to traditional all-night bracing therapy [26,27,28,29,30]. Overcorrection in the night orthosis is achieved through maximum lateral rotation in the direction opposite to the curvature, with simultaneous pressure on the apex of the curvature and an axillary abutment [15]. In the supine position, the spine is 5–7 cm longer and therefore allows for a larger correction area, which is of particular importance when utilizing the axillary abutment. Furthermore, the physiological shape of the spine is flattened in the supine position. This results in an enhanced ability to correct the major and minor curves (Figure 1) [31]. However, there is still an ongoing discussion regarding the superiority or non-inferiority of the Charleston Bending Brace compared to custom-made full-time braces such as the Chêneau brace. Several systematic reviews evaluated the available studies which compared the outcomes after treatment with nighttime or full-time braces [31,32,33]. Due to the retrospective nature of the majority of the studies, the evidence level was low, and no clear treatment recommendations emerged from the studies. The comparability of the studies is complicated not only by different methodical approaches and defined endpoints but also by the wide variety of patient factors that can influence the outcome. Therefore, it is important to assess which patient factors influence initial correction as well as medium-term outcomes.

The objective of this study was to evaluate the primary correction of the main curve in the Charleston Bending Brace based on a retrospective analysis of radiographs of patients who were treated solely with the nighttime brace. In particular, the impact of various factors on the correction of the major curve in AIS was investigated.

## 2. Materials and Methods

**Figure 2:**
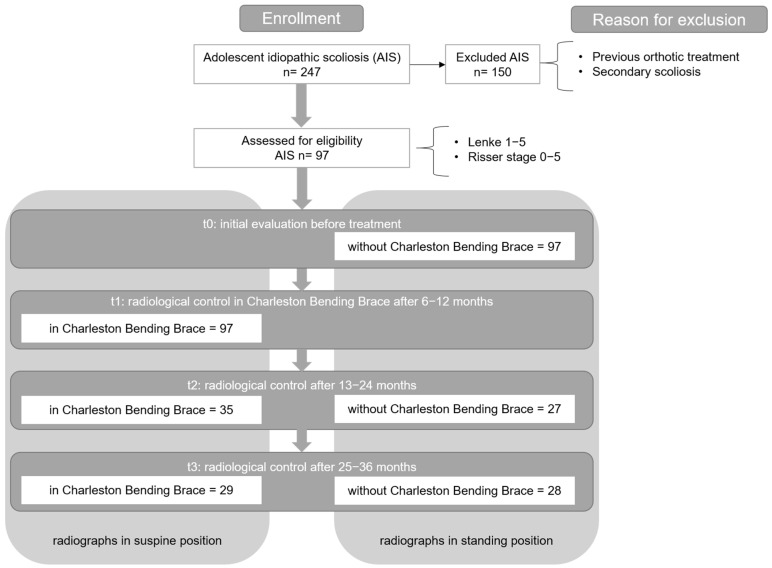
Inclusion and exclusion criteria including follow-up.

This study is a retrospective analysis of routine data and radiographic images of patients of the Orthopedic Clinic and Polyclinic of the Rostock University Medical Centre who were diagnosed with AIS between October 2010 and September 2020. Subsequently, follow-up assessments were conducted to complete the longitudinal course. A total of 247 cases were reviewed, and 97 patients met the inclusion criteria (AIS, Lenke curve type 1–5, Risser stage 0–5). Patients who had previously undergone orthotic treatment or who exhibited secondary scoliosis were excluded from this study (Figure 2). All patients included in this study were initially treated with the Charleston Bending Brace. All orthoses were individually custom-made by the same orthopedic technology team, and their fit was medically assessed by the same experienced pediatric orthopedic specialist.

The study was approved by the Ethics Committee of the University Rostock Medical Center (number: A 2020–0294).

As part of the initial diagnostic assessment, a comprehensive medical history was obtained, encompassing epidemiological data and a detailed clinical examination. Standardized radiographic imaging of the spine in a standing position (t0) in two planes was conducted to verify the AIS radiographically. The Cobb angle of the main and minor curves, the Lenke classification, the Risser stage, and finally, the rotation according to the Nash and Moe criteria were determined from the radiographs [34]. Figure 2 illustrates the inclusion and exclusion criteria, along with the number of patients assessed at each follow-up time point.

Apart from radiographs in the supine position in the Charleston Bending Brace for the majority of patients (t1–t3), an additional radiograph in the standing position without the brace was taken at t2 and t3 to monitor the curve progression for comparison with the initial angle (t0) to ensure that the curve had not deteriorated during treatment. Also, for the closing examination, patients were only radiographed in the standing position without the brace. In general, all in-brace radiographs were taken in the supine position, and all out-of-brace radiographs including pre-bracing images were taken in the standing position.

Preoperative radiographs were analyzed for each patient using the Cobb technique [3,7,35,36] to measure the curve pattern, and curves were categorized in accordance with the curve type, sex, the onset of menarche, the Lenke classification, Risser stages, and the Nash and Moe method to determine the relevant influencing factors.

### Statistical Analysis

The statistical analysis of the collected data was conducted using the software SPSS 27.0 (IBM Deutschland GmbH, Ehningen, Germany). The quantitative variables were described using the median, minimum (Min), maximum (Max), and the number of available observations (n). For the qualitative variables, the absolute and relative frequencies of the individual characteristics were reported. As part of the descriptive statistics, the data were tested for a normal distribution using the Shapiro–Wilk test. Normally distributed data were analyzed with the unpaired *t* test or ANOVA. When the data were not normally distributed, the Mann–Whitney U test and the Kruskal–Wallis test were subsequently used as non-parametric tests to examine possible significant differences regarding angle correction within the patient groups. The unpaired *t* test and the Mann–Whitney U test are suitable for comparing two patient groups, while ANOVA or the Kruskal–Wallis test, along with the subsequent Bonferroni post hoc test, was used for comparing at least three groups. The respective tests are indicated in the tables and figures. For the post hoc test, the Bonferroni-adjusted *p*-values for multiple comparisons were reported. A significance level of *p* < 0.05 was defined.

## 3. Results

During the observation period, a total of 247 patients with AIS were treated at our clinic, of whom 97 met the inclusion criteria for the study. The average age of patients was 12.8 ± 2.66 years.

Table 1 shows the baseline data of the entire cohort. Considering the entire patient population, the median initial major curve angle was 25.7°, with a maximum of 60.8° and a minimum of 10.7°. In patients with s-shaped scoliosis (43%), the angles of the major as well as the minor curves were assessed, and the values for the minor curve of this subgroup are also listed in Table 1.

### 3.1. Overall Curve Correction of the Major Curve During Follow-Up

Treatment with the Charleston Bending Brace resulted in a significant correction of the deformity. When in the supine position and gartered into the brace, the curvature of the major curve was significantly reduced by 74.2% from an initial median major curve of 25.7°; while standing, it reduced to 6.5° (Min 0°; Max 37.2°; *p* < 0.001 compared to Cobb angle at t0 with Wilcoxon test) when in the brace for six to twelve months (t1) after treatment with the Charleston Bending Brace commenced. Continuing the treatment for 13 to 24 months (t2) resulted in a more pronounced improvement of the initial angle to a median Cobb angle of 1.8° (Min 0°; Max 29.1°; *p* < 0.001 compared to Cobb angle at t0 with Wilcoxon test). This corresponded to a median 91.0% in-brace correction (worst −5.5%; best 100%). After 25 months (t3 = 25 to 36 months), improved Cobb angles deteriorated slightly in the in-brace radiographs to a median Cobb angle of 7.1° (Min 0°; Max 33.5°). However, the reduction in the Cobb angle at t3 was still significant (*p* < 0.001 compared to Cobb angle at t0 with Wilcoxon test), and the achieved overall in-brace correction amounted to 71.1% (worst −7.3%; best 100%) compared to t0.

Radiographs in the standing position without the brace also showed significant improvements in the curvature of the major curve, with 17.3% (*p* < 0.001) and 9.9% (*p* = 0.031) at t2 and t3, respectively. The slight deterioration that was observed between t2 and t3 for the correction while wearing the brace as well as in the improvement of the curvature without the brace could be due to the different patient populations assessed at t2 and t3. The patients at t3 might represent a subpopulation that required longer treatment due to confounding factors and were thus more difficult to treat.

Therefore, a detailed analysis of influencing factors such as the diagnosis of s-shaped scoliosis, gender, the Lenke classification, the Risser stage, the onset of menarche, and the rotation according to the Nash and Moe method on the percentage of achieved correction was performed for t1–t3. The patients were grouped accordingly.

### 3.2. Group-Specific Primary Curve Correction After t1 in the Brace

The primary in-brace curve correction was calculated as the percentage of improvement in the curvature of the spine while in the supine position and wearing the Charleston Bending Brace in the first year (t1) after the start of therapy compared to the curvature of the major curve in the standing position prior to treatment (Table 2). Depending on the group classification, the outcome differed significantly for some of the analyzed factors.

Table 2 shows that curve localization, the Lenke classification, and the Nash and Moe rotation were factors that significantly influenced primary in-brace curve correction. The analyses revealed that patients with lumbar major curves, Lenke 5, and Nash–Moe 1 benefited more from the treatment with the Charleston Bending Brace than patients in the other respective groups. While patients with Risser stage 2 seem to benefit most, the influence of the Risser stage on the outcome did not reach significance. Post hoc comparisons after overall analysis with the Kruskal–Wallis test (Table 2) demonstrated significant differences between Lenke 1 and 5 (*p* < 0.001, Bonferroni post hoc test) and Lenke 2 and 5 (*p* < 0.001, Bonferroni post hoc test). Also, patients with s-shaped scoliosis showed significantly lower in-brace correction. Accordingly, there was a statistical trend where the correction of the minor curve was lower than the correction of the major curve. However, that was only observed when all patients were included in the analysis. When only patients with s-shaped scoliosis were analyzed, there was no difference in the in-brace correction of the major and minor curves (*p* = 0.743, Mann–Whitney U test), as indicated by the percentages of 67.1% (major curve in s-shaped scoliosis) and 67.3% (minor curve).

### 3.3. Group-Specific Curve Correction During Follow-Up

**Figure 3:**
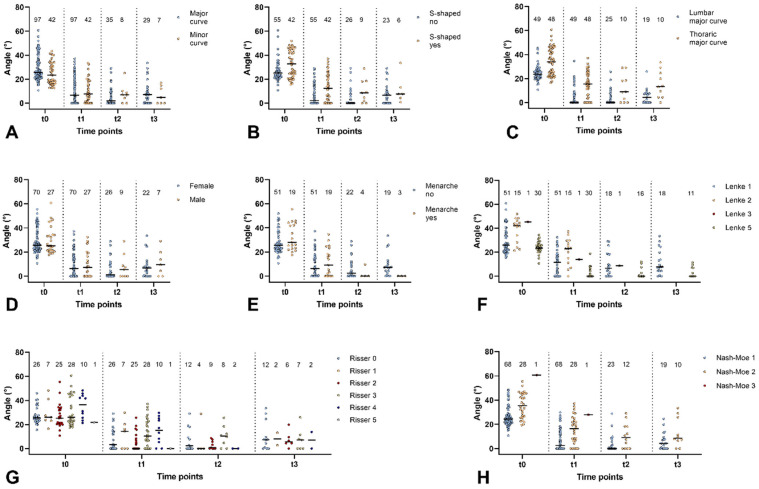
Cobb angle data from standing radiographs at t0 (pre-bracing) and from in-brace radiographs in the supine position for all follow-up time points (t1: 6–12 months, t2: 13–24 months, and t3: 25–36 months) during treatment with the Charleston Bending Brace. Data are presented as the distribution of individual values. The median is indicated by a black line, and the numbers on top of each column represent the sample size. The data are depicted separately according to the defined groups, as indicated in the legends of the graphs for (**A**) the major or minor curve, (**B**) the diagnosis of s-shaped scoliosis, (**C**) the curve type of the major curve, (**D**) gender, (**E**) the onset of menarche, (**F**) the Lenke classification, (**G**) the Risser stage, and (**H**) the Nash–Moe classification.

Figure 3 shows the influence of the different factors on the determined Cobb angles over time from t0 (in the standing position without the brace) up to t3 (all the latter in the supine position while wearing the brace). The graphs in Figure 3 illustrate the wide distribution of the individual Cobb angle values throughout the study and in the defined groups, but they still show that correction was achieved due to the intervention and was maintained for the entire follow-up period. Apart from the achieved correction in the major curve as described above, the percentage of correction of the minor curve was higher at t2 (median 70.5%; worst 3.4%; best 100%) and t3 (median 68.6%; worst 27.5%, best 100%), with significant reductions in the Cobb angle (t2: median 6.9; Min 0°; Max 25.0° and t3: median 4.8; Min 0°; Max 16.9°; Wilcoxon test *p* = 0.017 and *p* = 0.018 for t2 and t3 vs. t0, respectively). While Figure 3 depicts the individual Cobb angles in the different groups, statistical analysis was performed based on the percentage of in-brace correction to allow for the comparison of normalized data. Results showed that there were no significant differences in curvature correction between the major and minor curves (Figure 3A, Mann–Whitney U test: t2: 93.1% vs. 78.2%, *p* = 0.302 and t3: 70.5% vs. 68.6%, *p* = 0.969 for major vs. minor curve, respectively). There were also no significant differences due AIS being s-shaped or not (Figure 3B, Mann–Whitney U test: t2: 100% vs. 74.5%, *p* = 0.210 and t3: 76.1% vs. 61.1%, *p* = 0.276 for s-shaped scoliosis no vs. yes, respectively). The influence of curve localization on the percentage of correction was no longer significant at t2 (Mann–Whitney U test: 100% vs. 68.7%, *p* = 0.174 for lumbar vs. thoracic curve, respectively); however, at t3, there was a significantly higher in-brace correction in patients with lumbar scoliosis compared to thoracic scoliosis (Figure 3C, Mann–Whitney U test: 77.0% vs. 46.3%, *p* = 0.031 for lumbar vs. thoracic curve, respectively). Similarly, the greater benefit observed for patients with the onset of menarche (Figure 3E) was significant at t3 (Mann–Whitney U test: 65.7% vs. 100%, *p* = 0.014 for menarche no vs. yes, respectively), but not at t2 (Mann–Whitney U test: 88.5% vs. 100%, *p* = 0.389 for menarche no vs. yes, respectively). The Lenke classification corresponded best to the percentage of in-brace correction at both follow-up time points, with patients classified as Lenke 5 benefiting more than Lenke 1 patients (Figure 3F). Statistical tests of the percentage of correction showed significant differences between Lenke types 1 and 5 at t2 (Mann–Whitney U test: 67.8% vs. 100%, *p* = 0.008 for Lenke 1 vs. Lenke 5, respectively) and t3 (Mann–Whitney U test: 63.7% vs. 100%, *p* = 0.012 for Lenke 1 vs. Lenke 5, respectively) with regard to in-brace correction of the major curve. No significant differences in the in-brace results between groups at t2 and t3 were observed for sex (t2: *p* = 1.000; t3: *p* = 0.709, Figure 3D), the Risser stage (t2: *p* = 0.223; t3: *p* = 0.992, Figure 3G) and the Nash and Moe criteria (t2: *p* = 0.116; t3: *p* = 0.138, Figure 3H) in the respective non-parametric tests.

Despite the observed improvement in curve progression of the unimpeded major curve, i.e., when patients were radiographed in the standing position without the brace at t2 and t3, none of the studied factors showed a significant influence in the statistical analyses (Table 3).

The negative percentage values that were calculated from the out-of-brace radiographs indicate a progression in the major curve in some of the patients. At t2, only two patients (7.4%) showed a progression; at t3, the progression of the major curve was already detected in approximately a third of the out-of-brace radiographs (9/28 patients; 32.1%). Nearly half of the patients had an excellent initial in-brace correction (t1) of over 80%. When comparing the out-of-brace results at t3 from these patients to patients who had initial in-brace correction of 80% and less, it became apparent that 44% of patients in the lesser correction group showed a progression of the major curve compared to 17% in the group in which correction exceeded 80%. Importantly, progression ≥ 6° of the major curve only occurred in patients with lesser initial in-brace correction. However, these differences were not significant (Table 4). The values for the curve progression for the three patients with progression ≥ 6° were 7.5°, 8.3°, and 9.1°, respectively, and no curve progression ≥ 10° was observed.

## 4. Discussion

The present analysis investigated various aspects of the treatment of AIS with the Charleston Bending Brace. These included the primary in-brace correction of the main curve in the orthosis, the progression of the main and minor curves in the longitudinal course with and without the brace in place, and possible factors that could influence the longitudinal curve development. In addition to gender, the factors include the curve type according to Lenke’s classification, the curve localization, and the rotation according to the Nash and Moe classification. Furthermore, the existing growth reserve, which can be objectified by the Risser stage and, in girls, also by menarche, is a relevant factor [16,37,38].

The findings of the present study were evaluated in comparison with the results documented in the literature using the Chêneau corset, which is used as a 24 h brace.

It is recommended to initiate orthotic treatment when the main curve is between 20° and 25° in order to optimize the potential for achieving the desired outcome before the growth stops after adolescence [5,15,16]. Diverging from this recommendation, we also included two cases of AIS with a main curve below 20° in our study of the Charleston Bending Brace. In both cases, a deterioration of the clinical findings was observed within three months of the initial assessment, necessitating radiological follow-up. The radiographic images revealed a progression of the curve exceeding three degrees. The families of both children were already aware of pronounced scoliosis, which led to the decision being made in both exceptional cases to commence treatment at an earlier stage than would otherwise have been the case.

The current analysis revealed that the median primary angle at time t1 was corrected by 74.2% when wearing the brace. The localization of the curvature had a significant impact on the in-brace correction. The present cohort demonstrated a median in-brace correction in lumbar scoliosis of 100%, which was superior to the 53.9% median correction observed for thoracic curves. In a study by Korovessis et al. [39], the primary corrections of the major curves in the Chêneau corset were found to be 26% for thoracic curves, 34% for thoracolumbar curves, and 27% for purely lumbar curves after one year.

In a study on the Chêneau brace conducted by Hopf and Heine [40], the mean primary correction of the initial angle one year after brace treatment was 38% for thoracic single-arch AIS (n = 20) and 39.6% for single-arch lumbar scoliosis (n = 10). This discrepancy in correction can be attributed to anatomical factors. The rigid bony framework of the thorax presents a significant challenge for correction with a brace, particularly in comparison to the more mobile lumbar vertebral bodies, which are connected to the ribs, sternum, and spine. This difference also has implications for lung function [4,41]. The results of a study conducted by Minsk and Venuti [25] on the correction of the main curve in the Chêneau brace, which included 103 patients, also demonstrated a primary improvement in Cobb of only 31.5%. This result was also confirmed by Tsaknakis et al. [42]. Thus, our results conform to the literature that higher in-brace correction is usually achieved for lumbar scoliosis. The difference in the percentage values for the in-brace correction between our study and the literature on the Chêneau brace might be based on the different concepts of the brace types. By wearing the Charleston Bending Brace only at night, it is possible to overcorrect the curvature by bending the spine in the opposite direction when the patient is in the supine position. Our study showed that wearing the brace resulted in a perfect in-brace correction of 100% for more than one-third of the patients (36%). Similar high correction values for the Charleston Bending Brace were reported in previous studies [27,43]. However, bending the spine and thus the body in the opposite direction is not an option for patients when wearing a 24 h brace. Thus, initial in-brace correction is generally lower for full-time braces compared to nighttime brace due to the different concepts. This should be kept in mind when discussing the extent of the in-brace results.

The present study revealed substantial but non-significant differences in the primary correction at the time of the initial radiographs following the application of the brace (t1), particularly when analyzing the various Risser stages. While primary corrections of the main curve were found to be 84.9% in Risser stage 0 and 100% in Risser stage 2, lower primary improvements were recorded in stages 3–4 with the Charleston Bending Brace. In comparison to these results, Weiss und Werkmann [44] were able to demonstrate significant differences between the Risser stages with regard to primary correction in the Chêneau brace in a study comprising 81 patients.

It is generally known that patients with low Risser stages have a significantly greater growth potential and therefore a higher risk of AIS progression. Skeletal immaturity is considered an indicator for the start of brace therapy, as the bony parts of the spine are still considered malleable and the progression of AIS can be halted by guided growth [20,45]. In principle, this aspect could be substantiated by the present results. However, Risser stage 1 with a median primary correction of 51.0% and Risser 5 with a median curve improvement of 100% represent ‘outliers’. Risser stage 1 was only presented by seven patients in total. In this group, there were high initial angles paired with rotations according to Nash and Moe grades 2–3. In this respect, the curve correction was limited, even with the present low Risser 1. With regard to the result for Risser 5 with a median correction of 100%, it should be noted that this one case involved a lumbar curve of 21.9°, which could be completely corrected in the brace. The inclusion of the patient with Risser stage 5 was due to the fact that, although the iliac crest apophysis was already well ossified, menstruation had only started 1.5 years previously; therefore, it had to be assumed that there was still a potential growth reserve.

With regard to the influencing factor menarche on the in-brace correction of the main curve at t1, there was no significantly different primary correction in patients with menstruation compared to patients without menstruation. A study by Giorgi et al. [41] with 48 patients also showed no influence of the onset of menarche as found by us for the time of the first radiological control at t1.

When analyzing the different curve types in the present study, no significant difference in correction was found between the main and minor curves with the first radiographs after brace fitting (t1). However, single-arch AIS was corrected significantly better than s-shaped scoliosis, with the correction of the major and minor curves in s-shaped scoliosis being nearly identical at 67.1% and 67.3%.

Also, a comparison of the Lenke curve types and existing rotations according to Nash and Moe revealed significant differences for curve correction at t1. While a median primary correction of 100% was determined for Lenke 5 compared to the initial Cobb angle, this was significantly lower for Lenke 1 and 2 at 67.6% and 46.7%, respectively. This fact can be explained by the curve types themselves. Lenke 5 corresponds to the single-lumbar curve, which, due to its flexibility, can be straightened better than the thoracic single curve of Lenke 1 or the thoracic double curve of Lenke 2. With regard to the Nash and Moe rotation component, a significantly better primary correction (89.3%) was seen for Nash and Moe 1 in the Charleston Bending Brace than for Nash and Moe 2 (63.4%). The lower rotation at the beginning of the brace treatment had a positive effect on the curve in the frontal plane. A higher rotation component, especially in the thoracic region, is usually associated with a higher initial Cobb angle of the main curve. The extent of rotation is proportional to the extent of the main curve [46], meaning that a pronounced main curve is associated with a higher rotational component. The results of the present study demonstrated a better primary correction of the main curve of the AIS in the Charleston Bending Brace compared to the Chêneau brace [25,44,47]. In addition, up to one year after brace treatment, curve localization, Lenke classification, and the rotation component according to Nash and Moe proved to be significant influencing factors with regard to the primary correction of the main curve of the AIS. However, despite the higher in-brace correction of the nighttime brace, this was not necessarily associated with a better outcome in the long term when compared to full-time braces [27,43]. Still, Katz et al. reported that there was a positive correlation between the extent of in-brace correction and the orthosis’ ability to prevent curve progression. They observed that when initial in-brace correction with the Charleston Bending Brace exceeded 80%, the likelihood of a successful outcome was significantly higher [27]. This is in accordance with our data, which showed that none of the patients with an initial in-brace correction of more than 80% showed curve progression ≥ 6° in the out-of-brace radiographs at the 25–36-month follow-up.

In most cases, AIS develops progressively as a result of growth. In this respect, in addition to the evaluation of the primary correction of the main curve, the observation of the longitudinal course of therapy is of enormous importance to confirm therapy success, which depends on the different influencing factors [4,48,49,50].

At time points t2 and t3, median in-brace corrections of the main curve of 93.0% and 72.4% were determined. Without the brace, the mean improvements were 17.3% (t2) and 9.9% (t3). This was similar to the results observed after weaning with the Chêneau brace [40]. Since the difference to the initial angle is statistically significant in the medium term, we can conclude that the Charleston Bending Brace is suitable as the sole treatment option, considering the limitations of our study. The decrease in in-brace correction could be explained by the pubertal growth spurt, which influences the longitudinal growth of the spine and thus the development of the major and minor curves of the AIS. A deterioration of the initial angle is frequently observed due to rapid longitudinal growth [51]. The ultimate aim of orthotic treatment is to prevent significant progression [52]. The reduction in the correction of the main curve in the longitudinal course was also shown in the treatment of AIS in the Chêneau brace [39].

The changes in the main curve in the longitudinal course were not dependent on gender, the Risser stage, or the rotational component. However, there were significant differences for in-brace correction according to the Lenke classification. Lenke 5 patients had significantly better in-brace correction at time t3 than Lenke 1 patients. At time t3, curve localization and menarche also proved to be significant influencing factors on the in-brace correction of the main curve. The results at t3 show that the correction of lumbar AIS was more successful than that of thoracic malposition (60.2% vs. 81.3%), and patients who had already entered into their menstrual cycle demonstrated a significantly higher rate of correction for the main curve (100%) compared to those who were not menstruating (70%). The greater flexibility of the lumbar curve is well known. With regard to the difference in menarche, it should be noted that a significantly lower growth potential after the onset of menstruation and thus a lower progression of curve deformity can be assumed [16]. Our longitudinal results therefore contradict those of Giorgi et al. [53].

A reduction in the in-brace curve correction can also be observed over the longitudinal period for the AIS in the Chêneau brace [8,53]. Overall, the comparison of the longitudinal correction between the Charleston Bending Brace and the Chêneau brace appears to be possible only to a limited extent, as the time points of the radiological measurement of curve progression were different between the studies. Nevertheless, the Charleston Bending Brace demonstrates at least an equivalent degree of in-brace correction of the primary curve of the AIS at a follow-up period of 24 or 36 months. Consequently, it represents a promising alternative to a 24 h corset [27,53,54]. Apart from the potential benefits on curve progression in AIS, the study’s situation on compliance with regard to wearing behavior shows advantages in favor of the Charleston Bending Brace [26]. According to the literature, the so-called 24 h braces were not compliant in 40% of the treatments, i.e., less than 50% of the actual wearing time was used. In contrast, the use of the Charleston Bending Brace showed an incompliance rate of only 8% [28]. Finally, it is important to emphasize that individualized manufacturing to achieve the necessary overcorrection is essential for the success of the treatment.

## 5. Conclusions

From the present study it can be assumed that the Charleston Bending Brace represents a standalone treatment option for AIS. The Charleston Bending Brace demonstrated higher primary in-brace correction than reported values for the Chêneau brace; however, its long-term efficacy remains uncertain. Following a period of growth, it is reasonable to assume that the curve will progress, although the extent of this progression is likely to be minimal. The current findings demonstrate that treatment with the Charleston Bending Brace results in effective Cobb stabilization, thereby predicting a favorable prognosis with regard to the anticipated clinical evolution. Nighttime bracing in our study showed the best efficacy in patients with the Lenke 5 curve type and lumbar AIS.

## 6. Limitations

As the present study employed a retrospective design, there were differences between individual patients with regard to the exact measurement time points t1, t2, and t3 of the radiographs. Therefore, the time points are given as periods of several months instead of a fixed time point. A further limitation of this study is that only medium-term results were analyzed, with no data available after the completion of the entire treatment course, thus preventing a comprehensive assessment of the long-term results. Furthermore, it should be noted that the wearing behavior of the braces, which is not subject to precise control, represents a potentially limiting factor in the study and could have an influence on the results. It is important to consider the varying study designs when analyzing and comparing the literature. In addition to the size of the patient cohort, there may be slight differences in the study’s follow-ups, inclusion criteria, and methods. A major limitation with regard to the conclusion is the lack of the direct comparison between the Charleston Bending Brace and the Chêneau brace. Unfortunately, due to our patient population and their treatment options, it was not feasible to study a matched cohort for the Chêneau brace. Thus, we had to rely on data from the literature to evaluate whether the Charleston Bending Brace was as efficient as the Chêneau brace in preventing curve progression. However, the comparison of our results with those from the literature contains the risk of bias as there may be multiple variables, including the method of performing the full-spine radiography (supine or in standing position), the type of scoliosis and its rigidity, and finally, the lack of a direct comparison. While our results indicate that the Charleston Bending Brace can be a valuable alternative, not least because of reports of better compliance, the option of the “Charleston Bending Brace as the sole intervention” needs to be proven in prospective, randomized studies including long-term results. Such a study is the Bracing Adolescent Idiopathic Scoliosis (BASIS) study, a nighttime versus full-time bracing study in adolescent with idiopathic scoliosis [55]. Future results from the BASIS study are likely to allow for conclusions regarding the superiority or non-inferiority of the Charleston Bending Brace.

## Figures and Tables

**Table 1:** Baseline t0 patient demographics and clinical data with the respective curve values per defined group.

	No. of Patients	Percentage	Cobb Angle		
			Median	Maximum	Minimum
Major curve	97	100	25.7°	60.8°	10.7°
Minor curve	42	43.3	23.45°	43.4°	12.6°
Major curve in subgroups:					
S-shaped: no	55	56.7	25.0	60.8	10.7
S-shaped: yes	42	43.3	32.8	51.9	15.5
Lumbar major curve	49	50.5	23.5°	45.4°	10.7°
Thoracic major curve	48	49.5	33.9°	60.8°	16.5°
Female	70	72.2	25.8°	55.5°	10.7°
Male	27	27.8	25.3°	60.8°	17.7°
Lenke 1	51	52.6	26.0°	60.8°	15.5°
Lenke 2	15	15.5	42.2°	51.9°	21.4°
Lenke 3	1	1.0	44.4°	44.4°	44.4°
Lenke 5	30	30.9	23.6°	34.4°	10.7°
Risser Stage 0	26	26.8	25.0°	46.1°	15.5°
Risser Stage 1	7	7.2	26°	48.4°	16.5°
Risser Stage 2	25	25.8	25.3°	55.5°	10.7°
Risser Stage 3	28	28.9	26.05°	60.8°	17.4°
Risser Stage 4	10	10.3	36.45°	48.4°	21.3°
Risser Stage 5	1	1.0	21.9°	21.9°	21.9°
Nash–Moe 1	68	70.1	24.3°	48.4°	10.7°
Nash–Moe 2	28	28.9	35.5°	55.5°	19.4°
Nash–Moe 3	1	1.0	60.8°	60.8°	60.8°
Menarche: no	51	72.9	25.4°	51.9°	10.7°
Menarche: yes	19	27.1	28.1°	55.5°	17.4°

To distinguish the different categories, a background color has been chosen.

**Table 2:** Comparison of the percentage of primary in-brace curve correction (t1) according to potential influencing factors. Statistical analysis was performed with either * the Mann–Whitney U test or ** the Kruskal–Wallis test. Subgroups containing only one patient were not included in the statistical analysis.

	n	Median in %	Worst in %	Best in %	*p*-Value
Major curve	97	74.2	12.9	100.0	*p* = 0.066 *
Minor curve	42	67.3	2.3	100.0	
Major curve compared in subgroups:					
S-shaped: no	55	91.6	12.9	100.0	*p* = 0.015 *
S-shaped: yes	42	67.1	21.7	100.0	
Lumbar major curve	49	100.0	18.7	100.0	*p* < 0.001 *
Thoracic major curve	48	53.9	12.9	100.0	
Female	70	71.4	13.3	100.0	*p* = 0.443 *
Male	27	74.8	12.9	100.0	
Lenke 1	51	67.6	12.9	100.0	*p* < 0.001 **
Lenke 2	15	46.7	21.7	100.0	
Lenke 3	1	69.2	69.2	69.2	
Lenke 5	30	100.0	45.3	100.0	
Risser 0	26	84.9	12.9	100.0	*p* = 0.067 **
Risser 1	7	51.0	37.7	100.0	
Risser 2	25	100.0	29.8	100.0	
Risser 3	28	65.6	13.3	100.0	
Risser 4	10	54.7	28.9	100.0	
Risser 5	1	100.0	100.0	100.0	
Nash–Moe 1	68	89.3	13.3	100.0	*p* = 0.005 *
Nash–Moe 2	28	63.4	12.9	100.0	
Nash–Moe 3	1	53.9	53.9	53.9	
Menarche: no	51	71.1	18.7	100.0	*p* = 0.731 *
Menarche: yes	19	71.7	13.3	100.0	
* Mann–Whitney U test					
** Kruskal–Wallis test					

To distinguish the different categories, a background color has been chosen.

**Table 3:** Comparison of the percentages of out-of-brace curve correction at t2 and t3 according to the potential influencing factors. Statistical analysis was performed as indicated. Subgroups containing only one patient were not included in the statistical analysis.

	N t2	t2: Median [Worst; Best] in %	*p*-Value	N t3	t3: Median [Worst; Best] in %	*p*-Value
Major curve	27	17.3 [−22.1; 50.1]		28	9.9 [−46.9; 100.0]	
Major curve compared in subgroups:						
Lumbar major curve	16	19.2 [−9.2; 47.7]	*p* = 0.622 ^#^	16	9.3 [−46.9; 100.0]	*p* = 0.708 ^#^
Thoracic major curve	11	17.3 [−22.1; 50.1]		12	14.7 [−35.2; 51.8]	
Female	16	16.6 [−9.2; 50.1]	*p* = 0.823 ^#^	20	9.3 [−46.9; 100.0]	*p* = 0.916 ^#^
Male	11	17.3 [−22.1; 42.0]		8	14.7 [−35.2; 66.0]	
Lenke 1	16	19.2 [−22.1; 50.1]	*p* = 0.757 ^#^	17	10.5 [−46.9; 100.0]	*p* = 0.647 ^#^
Lenke 2	1	22.9	(Lenke 1 vs. 5)			
Lenke 5	10	16.3 [2.5; 31.7]		11	9.3 [−13.3; 66.0]	
Risser 0	9	16.2 [2.5; 47.7]	*p* = 0.546 **	9	10.5 [−21.8; 66.0]	*p* = 0.799 ^##^
Risser 1				3	9.3 [−12.4; 37.5]	
Risser 2	9	22.0 [9.2; 50.1]		8	23.7 [1.2; 38.3]	
Risser 3	6	24.2 [7.8; 38.6]		6	3.5 [−46.9; 100.0]	
Risser 4	3	4.5 [−22.1; 28.9]		2	−12.9 [−31.5; 9.3]	
Nash–Moe 1	18	16.8 [−22.1; 47.7]	*p* = 0.492 ^#^	15	19.1 [−35.2; 100.0]	*p* = 0.201 *
Nash–Moe 2	9	24.8 [4.5; 50.1]		13	9.3 [−46.9; 24.1]	
Menarche: no	11	8.7 [−9.2; 47.7]	*p* = 0.451 ^#^	16	0.4 [−46.9; 51.8]	*p* = 0.061 ^#^
Menarche: yes	5	28.9 [4.5; 50.1]		4	22.9 [9.3; 100.0]	
Parametric tests:		^#^ unpaired *t* test or ^##^ ANOVA				
Non-parametric tests:		* Mann–Whitney U test or ** Kruskal–Wallis test				

To distinguish the different categories, a background color has been chosen.

**Table 4:** Patients with progression in out-of-brace radiographs at t3 according to initial in-brace correction.

	N t3	Number of Patients with Progression in Out-of-Brace Radiograph at t3 (%)	Number of Patients with Progression ≥ 6° in Out-of-Brace Radiograph at t3 (%)
Initial in-brace correction at t1:			
≤80% (n = 55; 56.7%)	16	7 (43.8%)	3 (18.8%)
>80% (n = 42; 43.3%)	12	2 (16.7%)	0 (0%)
*p*-value (Fisher’s exact test)		*p* = 0.223	*p* = 0.238

## Data Availability

The data presented in this study are available upon request from the corresponding author. The data are not publicly available but can be obtained from the Department of Clinical Research at the Orthopedic Department of the University Medicine Rostock if required.
